# Applying human-centered design to maximize acceptability, feasibility, and usability of mobile technology supervision in Kenya: a mixed methods pilot study protocol

**DOI:** 10.1186/s43058-020-00102-9

**Published:** 2021-01-07

**Authors:** Noah S. Triplett, Sean Munson, Anne Mbwayo, Teresia Mutavi, Bryan J. Weiner, Pamela Collins, Cyrilla Amanya, Shannon Dorsey

**Affiliations:** 1 Department of Psychology, University of Washington, Guthrie Hall 119A, Box 351525, Seattle, WA 98195 USA; 2 Department of Human Centered Design & Engineering, University of Washington, 428 Sieg Hall, Box 352315, Seattle, WA 98195 USA; 3 Department of Psychiatry, University of Nairobi, P.O. Box 19676, Nairobi, 00202 Kenya; 4 Department of Global Health, University of Washington, Harris Hydraulics Laboratory, 1510 San Juan Road, Seattle, WA 98195 USA; 5 Department of Health Services, School of Public Health, University of Washington, Box 357965, Seattle, WA 98195 USA; 6 Department of Psychiatry and Behavioral Sciences, University of Washington, 1959 NE Pacific Street, Box 356560, Seattle, WA 98195 USA; 7 Research Department, Ace Africa Kenya, P.O. Box 1185, Bungoma, 50200 Kenya

**Keywords:** Human-centered design, Task-shifting, Supervision, Mobile technology

## Abstract

**Background:**

Although research continues to support task-shifting as an effective model of delivering evidence-based practices (EBPs), little scholarship has focused how to scale up and sustain task-shifting in low- and middle-income countries, including how to sustainably supervise lay counselors. Ongoing supervision is critical to ensure EBPs are delivered with fidelity; however, the resources and expertise required to provide ongoing supervision may limit the potential to scale up and sustain task shifting. Opportunities may exist to leverage mobile technology to replace or supplement in-person supervision in low-resource contexts, but contextual variables, such as network connectivity and lay counselor preferences surrounding mobile technology, must be examined and considered when designing and implementing mobile technology supervision.

**Methods:**

This study builds from an existing randomized trial in Kenya, wherein teachers and community health volunteers have been trained to provide trauma-focused cognitive behavioral therapy as lay counselors. The study will use an iterative and mixed methods approach, with qualitative interviews and a Human-Centered Design (HCD) workshop informing a non-randomized pilot trial. Semi-structured interviews will be conducted with lay counselors and supervisors to understand how mobile technology is currently being used for supervision and determine the barriers and facilitators to mobile technology supervision. Data from these interviews will inform an HCD workshop, where lay counselors and supervisors “re-design” supervision to most effectively leverage mobile technology. Workshop participants will also participate in focus groups to gather perceptions on the use of HCD techniques. The final outcome of the workshop will be a set of refined workflows, which will be tested in a mixed method, nonrandomized pilot with newly trained lay counselors and existing supervisors. The pilot trial will evaluate the acceptability, feasibility, and usability of mobile technology supervision through self-report questionnaires as well as perceptions of effectiveness through qualitative interviews with a subset of lay counselors and all supervisors.

**Discussion:**

This study will provide a launching point for future research on supervision and methods to engage stakeholders to design and tailor interventions and implementation supports to fit low-resourced contexts.

**Trial registration:**

The parent trial from which this study builds was registered on ClinicalTrials.gov on August 9, 2017 (NCT03243396).

**Supplementary Information:**

The online version contains supplementary material available at 10.1186/s43058-020-00102-9.

Contributions to the literature

- Supervision is an important implementation strategy to support task-shifting of evidence-based practices for mental health problems; however, the resources required to provide in-person supervision may limit the ability to scale up and sustain task shifting.
- Human-centered design offers a framework to engage lay counselors and supervisors to “redesign” supervision to leverage mobile technology, thereby increasing the sustainability of supervision and task-shifting.
- This study will provide preliminary data on the use of mobile technology to supervise lay counselors while also evaluating the use of human-centered design methods in global mental health and implementation science.

## Background

Mental health disorders are among the leading contributors to the global burden of disease [1]. While approximately 80% of the world’s population lives in low- and middle-income countries (LMIC), most of the world’s mental health resources (including human resources) are in high-income countries [2]. As a result, a mental health treatment gap exists where relatively few individuals with mental health disorders in LMIC receive needed mental health care [3]. One strategy for addressing the workforce shortages that contribute to the mental health treatment gap is task-shifting, in which lay counselors (e.g., community members, health workers) without formal mental health training or experience are trained and supported to deliver psychological interventions [4]. A growing body of evidence [5–9], including a Cochrane Review [10], supports the effectiveness of task-shifting to deliver evidence-based practices (EBPs) for mental, neurological, and substance use disorders in LMIC. Although research continues to support task-shifting as an effective model of delivery, little research has focused on how to scale up and sustain task-shifting in LMIC, including how to sustainably supervise lay counselors [11].

Research in high-income countries [12, 13] and growing evidence in LMIC [14] highlights that ongoing supervision is an important implementation strategy to ensure EBPs are delivered with fidelity (i.e., as intended by intervention developers [15]). However, the resources and expertise required to provide ongoing supervision to lay counselors are factors that limit the potential to scale up and sustain task shifting. The cost of in-person supervision has been a challenge for EBP delivery in the USA [16, 17]. This challenge may be amplified with task-shifting in lower-resourced contexts, such as LMIC, where funding is low and trained mental health providers who can serve as supervisors are more limited in number. Further, to support lay-counselors in rural areas, supervisors may need to travel long distances to conduct in-person supervision, with inclement weather adding to transportation costs and time (e.g., rainy seasons may cause travel cost and time to increase in many places). Opportunities may exist to leverage mobile technology to replace or supplement in-person supervision in low-resource contexts, which could reduce costs and improve the sustainability of supervision. A small body of literature has examined how technology can be used across a variety of implementation strategies in LMIC, including as a tool to support supervision during in-person meetings [18]. Anecdotally, we also know of several projects where mobile technology has emerged as an unplanned supervision and implementation strategy. The extent to which mobile technology could feasibly replace in-person supervision meetings is unknown. Contextual variables, such as limited network connectivity or lay counselor and supervisor preferences surrounding mobile technology, must be examined and considered when designing and implementing mobile technology supervision practices.

The present article outlines an iterative, mixed-methods study that engages lay counselors and supervisors to design and test a method of using mobile technology to replace in-person supervision for lay counselors in Kenya. We seek to gather contextual knowledge and anticipate challenges with scaling up mobile technology supervision, thereby optimizing its acceptability, feasibility, and usability. Guided by human-centered design, we involve supervisors and lay counselors throughout the research process to identify potential barriers and generate solutions to using mobile technology to provide supervision. Study findings may provide information on how mobile technology can be utilized to provide clinical supervision and facilitate other implementation strategies across a variety of low-resource settings and interventions.

### Study aims

We aim to optimize and evaluate the acceptability, feasibility, and usability of mobile technology to conduct supervision and support lay counselors. We engage lay counselors and supervisors to garner local expertise, ownership, and contextual understanding through the following aims:

- **Aim 1:** To investigate ways mobile technology is currently being used to support supervision and identify barriers and facilitators of mobile technology supervision;
- **Aim 2:** To engage stakeholders to redesign supervision processes to most effectively utilize mobile technology;
- **Aim 3:** To evaluate the acceptability, feasibility, and usability of mobile technology supervision, as well as perceptions of effectiveness in a pilot trial.

## Methods

### Design overview

**Fig. 1:**
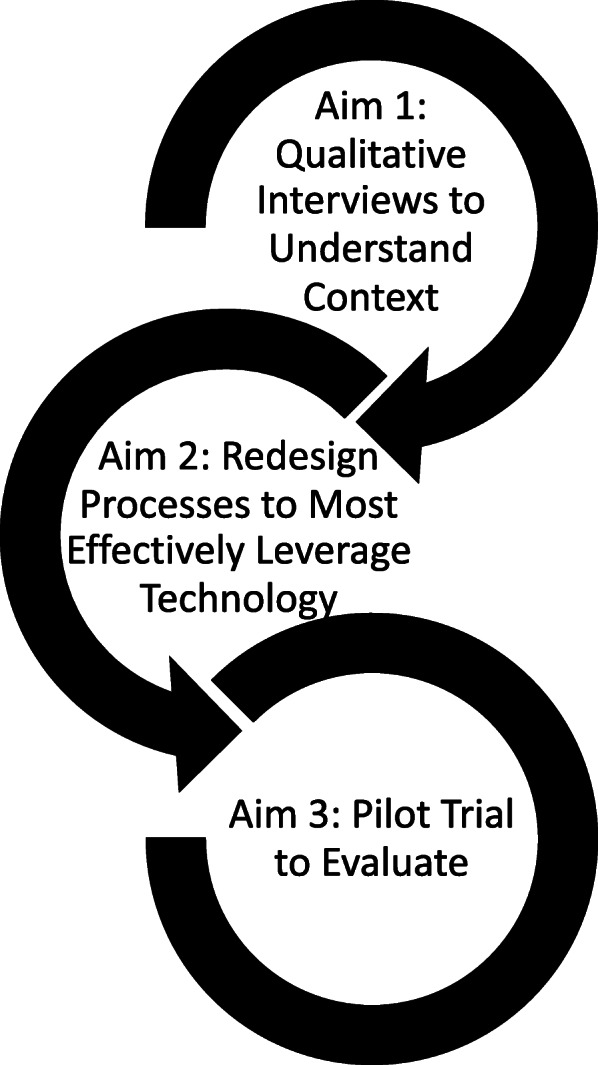
Study approach

This study will use an iterative and mixed methods approach, with qualitative interviews (*N* = 31) and a Human-Centered Design (HCD) workshop informing a non-randomized pilot trial (*N* = 37). Our goal is to understand and optimize the acceptability and feasibility of mobile technology supervision. For reporting, we follow the Lancaster and Thabane guidelines for reporting non-randomized pilot and feasibility studies [19], which advocates for the adapted use of the Consolidated Standards of Reporting Trials (CONSORT [20]; Additional file 1). This trial is situated within a larger stepped wedge cluster randomized trial that examines the implementation of a locally adapted version of trauma-focused cognitive behavioral therapy, called Pamoja Tunaweza, in Bungoma, Kenya [Building and Sustaining Interventions for Children (BASIC); see [21] for protocol]. Throughout the trial, we engage participants to anticipate challenges with using mobile technology to conduct supervision and propose strategies to overcome them. These strategies are refined and tested in a non-randomized pilot trial (Fig. 1). By drawing from local expertise to inform implementation support, we hope to garner local ownership and contextual understanding.

### Setting and participating sites

This trial builds on a NIMH-funded cluster randomized controlled trial in the area surrounding Bungoma, Kenya, “Building and Sustaining Interventions for Children (BASIC): Task-Sharing Mental Health Care in Low-Resource Settings” [21]. BASIC is a collaboration between researchers at Duke University, the University of Washington, and Kenyan partners at Ace Africa. BASIC aims to test the effectiveness and implementation of trauma-focused cognitive behavioral therapy (TF-CBT) [22] delivered by lay counselors in two government-supported systems: education (via teachers) and health (via community health volunteers [CHVs]). BASIC utilizes an 8-session version of TF-CBT (“*Pamoja Tunaweza*”), which was adapted by the Ace Africa supervisors and counselors for cultural relevance and acceptability. Presently, BASIC has trained 150 lay counselors (75 teachers and 75 CHVs). Lay counselors work together in groups of 3 to provide the treatment in a group-based format and are trained and supervised by 7 Kenya-based supervisors. Supervisors are Ace Africa employees who were previously trained (and subsequently delivered) the treatment in a randomized controlled trial [23] that preceded the current BASIC trial. Supervision has included face-to-face meetings with lay counselor groups and ad hoc mobile phone communications. Face-to-face supervision has been costly and time intensive. Mobile technology, including SMS and WhatsApp, emerged as a supervision and support strategy for some counselors, but the extent to which mobile technology is used and can be systematically implemented to support supervision is unknown.

### Conceptual framework/approach

**Fig. 2:**
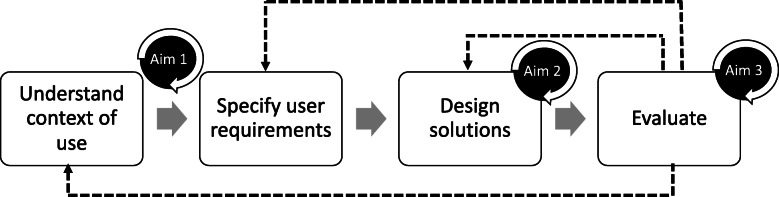
Interaction design foundation framework

Human-centered design (HCD) is a set of principles and procedures intended to make products (including interventions) more accessible and effective by grounding their development in the needs and preferences of those who will ultimately use them [24]. Though HCD originated in the context of software development, the principles of stakeholder-preference and involvement are widely applicable. A common element of HCD approaches is to gather stakeholder feedback through simulated examples (“prototypes”), which are designed to elicit concerns and behaviors across the continuum of implementation [25]. This feedback can then be considered to tailor interventions and implementation strategies to be acceptable or appropriate, two key determinants of implementation success [15]. There has been increased application of HCD to psychosocial [26] and global health [27] intervention development and implementation [28, 29]; however, HCD is just beginning to be applied to global mental health (see [30] for an example). HCD may be particularly useful for complex service delivery environments, where resource availability is dynamic and where interventions require adaptation to meet systems, resource, and other contextual specificities. Included within this context is mental health service delivery, given the nuanced contextual and cultural factors that must be considered to successfully implement psychosocial interventions in global settings. By incorporating HCD into the design and implementation of mobile technology supervision, researchers may be able to incorporate the needs and preferences of supervisors and counselors at each step of development, refinement, and implementation, thereby improving acceptability, appropriateness, and eventual sustainability. The Interaction Design Foundation Framework (see Fig. 2) [31] provides a lens through which the local context and expertise can be considered to develop and implement mobile technology supervision.

### Overview of study aims

Aim 1 will use qualitative research and HCD methods to engage supervisors and lay counselors to understand how mobile technology is currently being used for supervision. This corresponds to “understand context of use” and “specify user requirements” within the Interaction Design Foundation Framework (see Fig. 1) [31]. Interviews will elicit perceived benefits/disadvantages and barriers/facilitators of mobile technology supervision. Although we focus on designing for a specific context and need, we anticipate that these interviews will also form the foundation of knowledge that can be transferred to the scale up of mobile technology supervision across a variety of interventions and contexts, including with providers in the USA. Findings may also provide insight into how mobile technology can be used to facilitate the use of other implementation strategies in lower-resourced settings.

The goals of Aim 2 are to collaboratively “re-design” supervision to most effectively leverage mobile technology by (1) facilitating discussion and brainstorming of potential workflows (i.e., sequence of processes or actions lay counselors and supervisors would undertake) to enable mobile technology supervision and (2) selecting and refining workflows, such that a final set of 3 workflows is collaboratively chosen and refined to guide implementation in the Aim 3 pilot trial. This corresponds to “design solutions,” within the Interaction Design Foundation Framework (see Fig. 1) [31]. Drawing on the results of Aim 1, we seek to design solutions for the identified barriers and capitalize on perceived potential benefits of mobile technology supervision. As with Aim 1, Aim 2 draws from HCD techniques to ensure strategies match counselors’ and supervisors’ needs and preferences. We will also conduct focus groups with lay counselors and supervisors to gather their perceptions on the use of HCD techniques. Questions will focus on counselors’ and supervisors’ experience participating in HCD activities, with attention to the settings in which HCD techniques were developed and largely have been used (e.g., high-income country, western cultural norms).

We will use the workflows from Aim 2 to guide implementation of mobile technology supervision in Aim 3. Mobile technology supervision will be tested through a mixed method, nonrandomized pilot with 30 newly trained lay counselors and all interested supervisors. This corresponds to “evaluate,” within the Interaction Design Foundation Framework (see Fig. 1) [31]. We will evaluate the acceptability, feasibility, and usability of mobile technology supervision through self-report questionnaires. A subset of the 30 participating lay counselors and all participating supervisors will also participate in qualitative interviews to gather more information on their experience using mobile technology to receive or conduct clinical supervision, including any perceived benefits and disadvantages of the approach. Interviews will also focus on perceived effectiveness of mobile technology supervision.

### Aim 1: investigate ways mobile technology is currently being used to support supervision and identify barriers and facilitators of mobile technology supervision

#### Participants

Participants will include 24 (12 teachers and 12 CHVs) of the up to 150 lay counselors from BASIC who have already begun delivering TF-CBT as part of the parent trial and all interested supervisors. Twelve participants are often considered sufficient for saturation [32], but if new themes relevant to our aims emerge, we will increase enrollment. Lay counselor participants will be recruited via a purposeful sampling approach [33] to balance those who use mobile technology frequently, an average amount, and infrequently or rarely. The supervisors, being familiar with lay counselors that they are supervising, will be asked to rate lay counselors on how frequently they use mobile technology within their work. The ratings will be done with a 1-7 Likert-type scale that includes specific behavioral anchors. “Extreme” users—those using mobile technology with high frequency or rarely—may more easily illustrate the behaviors and needs of a population [34]. As such, 1/3 of participating counselors (8/24) will be high-frequency users of mobile technology. One third (8/24) will use mobile technology rarely or not at all. The remaining third will consist of average frequency users, allowing participation across the full range of user needs [35]. Our only exclusion criterion is that lay counselor and supervisor participants must have phones that are WhatsApp compatible. Trained local interviewers will obtain informed consent from all participants at the time of enrollment.

#### Procedure

Counselors and supervisors will participate in in-person, semi-structured interviews. Interviews will be conducted by a local interviewer in the language of the participant’s choosing (i.e., Kiswahili or English). Each interview will last approximately 1 h. In concordance with HCD [34], interviews will begin broadly, inquiring into work and values surrounding counseling. Questions will then become more tailored to supervision and the use of technology. Questions will solicit information on the extent of mobile technology use (including which applications or mediums of communication are typically used). We will also gather opinions on typical supervision practices as well as perceived barriers and facilitators of mobile technology supervision. Beyond traditional qualitative semi-structured interview questions, interviewers will employ HCD techniques, such as “scenarios of use” [36] to ground participant responses in hypothetical scenarios. Interviews will be recorded with participant permission; audio recordings and notes will be retained for qualitative analysis.

#### Analysis

Recordings from interviews will be transcribed and identifying information removed. Analysis will follow Braun & Clarke’s (2006) six-phase framework for thematic analysis [37]. Transcripts will be coded in NVivo [38] by researchers in the USA. Kiswahili interviews will be translated by a member of the research team. Trained coders will review transcripts, meet to identify potential codes, and produce an initial codebook to be subsequently refined. After finalizing a codebook, all reviewers will independently code. Consensus will be reached through group dialogue [39]. Emerging themes and coded data will be reviewed to extract *key insights* from each theme. Key insights are an HCD concept that reframes core themes in terms of specific problems, strengths, or processes that emerged from interviews [34]. For example, a key insight from interviews regarding the ease of using mobile technology when travel time is limited may be, “recording video-recorded role plays of counseling sessions and transmitting over WhatsApp may be helpful when in-person supervision is not possible.” For each insight, one or more *opportunity area(s)* will be developed that translates insights into opportunities by posing “how might we” questions. For example, an insight might be turned into “how might we send video-recorded role plays when mobile network connection is limited?” These key insights and opportunity areas will be used to facilitate discussion and brainstorming in Aim 2. In Table 1, we include additional example key insights that could arise, along with example opportunity areas. These examples are only to demonstrate how key insights translate into opportunity areas. We cannot know in advance what participants might identify.

**Table 1:** Example key insights, opportunity areas, and solutions and workflows

Example key insights	Example opportunity areas	Example solutions/workflows
Sending video-recorded role plays over WhatsApp may be helpful when in-person supervision is not possible	How might we structure video role playing to ensure maximum benefit?	Lay counselors will conduct role plays together; Supervisors will communicate what content they would like role played before.
WhatsApp use may be limited in communities without strong network connection	How might we build workarounds to ensure messages can be sent in poorer connection areas?	The lay counselor with best connection at home or on their commute will be tasked with sending the role play asynchronously
Counselors often turn off their data connection when not in use	How might we ensure counselors can access data when necessary without spending data when not necessary?	Lay counselors and supervisors will pre-select times during each day where they plan to turn on data and send communications

### Aim 2: engage stakeholders to redesign supervision processes to most effectively mobile technology

#### Participants

All participants from Aim 1 will be invited to participate in Aim 2. There are no additional exclusion criteria for Aim 2 beyond that of Aim 1—that lay counselor and supervisor participants must have phones that are WhatsApp compatible.

#### Procedures

Lay counselors and supervisors will convene for a retreat after Aim 1 interviews have been completed and analyzed. Participants will receive transportation and refreshments (e.g., snack or tea). A Kenyan member of the research team will orient participants to the goals (i.e., develop workflows to facilitate supervision via mobile technology), present findings from Aim 1, and facilitate brainstorming and development of strategies with the counselors and supervisors. Up to four *key insights* and *opportunity areas* from Aim 1 will be shared with counselors and supervisors as a means of member checking and for further refinement. After discussing findings, HCD methods will be used to facilitate a “co-creation session” with lay counselors and supervisions. Participants will brainstorm workflows for each *opportunity area*. The *opportunity areas* developed in Aim 1 will be posted on separate sheets of large paper or posterboard to facilitate co-creation of different workflows. Participants will first be asked to collectively generate a list of barriers associated with their area then directed to begin brainstorming *all* potential solutions to overcome barriers.

After the group has brainstormed about each opportunity area, participants will be randomly assigned into groups of 5 participants (4 lay counselors and one supervisor). Each group will be assigned one *key insight* and corresponding *opportunity area* then review all barriers and solutions that have been generated. Each group will then select a solution (or multiple solutions) and create one workflow of how their chosen solution(s) may be integrated into their current supervision structure and processes. Ultimately, each group will develop one workflow in response to one *key insight* and corresponding *opportunity area.* Workflows will include specific *activities* (i.e., a single, logical step in a process), *actions* (i.e., an action that accomplishes an activity), and *transitions* (i.e., movement from one activity and action to another) [40]. For instance, one group may focus on “limited network connection” and develop a comprehensive workflow for recording role plays where they practice (e.g., school) and sending to the supervisor *at a different time/place with better connection*. Another group may develop a workflow that coordinates when lay counselors will plan to turn on data to send and receive updates via WhatsApp from their supervisors. In Table 1, we include additional example solutions alongside corresponding *key insights* and *opportunity areas*. These examples are only to demonstrate how key insights translate into solutions. We cannot know in advance what participants might identify.

Groups will share their workflows with the larger group to gather initial feedback. In presenting their workflows, the group will be instructed to *walkthrough* carrying out the workflow [36]. After each team has presented their workflows, the larger group will discuss each workflow and ways to refine/optimize it. Discussion will also focus on how workflows could be adapted to address multiple opportunity areas. Counselors and supervisors will anonymously vote for their top 3 most feasible workflows. The 3 strategies that earn the most votes will be refined and retained to inform implementation in Aim 3.

After the retreat, participants (lay counselors and supervisors) will take part in focus groups to gather perceptions on the use of HCD techniques. Lay counselors will be randomly assigned into two focus groups, and supervisors will be convened separately. Questions will focus on counselors’ and supervisors’ experience participating in HCD activities, with attention to the settings in which HCD techniques were developed and largely have been used (e.g., high-income country; western cultural norms). Although HCD is promoted widely, to our knowledge, there have been no studies on the acceptability or perceptions of HCD techniques in LMIC.

#### Analysis

Qualitative analyses will follow the same thematic analysis procedure described in Aim 1.

### Aim 3: evaluate the acceptability, feasibility, and usability of mobile technology supervision, as well as perceptions of effectiveness in a pilot trial

#### Participants

Participants will be lay counselors recently trained in TF-CBT who have not started delivering TF-CBT as part of the parent trial (*N* = 30; 15 teachers; 15 CHVs) and all interested supervisors. This sample size was selected because it is the size of one cluster in the parent cluster-randomized trial [21]. We will purposefully select lay counselors nested under each interested supervisor. Exclusion criteria include those from Aims 1 and 2 (i.e., must have WhatsApp compatible phones) with an additional exclusion criterion of participation in Aims 1 and 2. Trained local interviewers will obtain informed consent from all participants at the time of enrollment.

#### Procedure

Workflows generated in Aim 2 will be developed into formal implementation guidance in the form of standard operating procedures (SOPs). The SOPs will be developed in collaboration with participating supervisors and delineate how supervision will be carried out via mobile technology. Supervisor participants will receive training on the SOPs during weekly calls and protocol review.

**Fig. 3:**
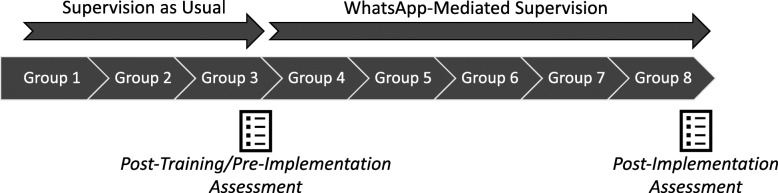
Pilot trial design

The nonrandomized trial of mobile technology supervision will occur when these newly trained counselors begin TF-CBT delivery, as part of their participation in the parent trial. At the beginning of TF-CBT delivery, supervisors will conduct supervision as usual (i.e., in-person with usual technology support) for the first 3 weeks of the 8-session TF-CBT protocol [21]. During the planning phase of this trial, supervisors expressed reservations about beginning exclusive mobile technology supervision at session 1. Supervisors wished to continue with some in-person supervision and more limited reliance on mobile support for the first 3 sessions. After session 3, supervisors will switch to mobile technology supervision for sessions 4–8. Supervisors will conduct abbreviated, in-person trainings in the mobile technology supervision protocol with lay counselors during week 3, before mobile technology supervision begins in week 4. Throughout the mobile technology period, supervisors will be able to see or hear lay counselors practice TF-CBT techniques and plan for upcoming groups similar to in-person supervision, just via mobile technology. If a safety concern arises about a new counselor’s ability or about a specific child, the mobile technology protocol will be overridden to ensure child safety. Supervisors and lay counselors will complete measures of acceptability and feasibility of supervision as usual immediately preceding switching to mobile technology supervision. Supervisors and lay counselors will complete measures of mobile technology supervision acceptability and feasibility, as well as a measure of usability, after completing the TF-CBT protocol (Fig. 3). Frequency of supervision contacts and any protocol deviations (i.e., in-person supervision meetings) will also be tracked.

A sub-sample of lay counselors (*N* = 12; 6 teachers; 6 CHVs) and all participating supervisors will participate in semi-structured interviews following the trial. Qualitative interview participants will be randomly selected from all Aim 3 participants. If differences emerge by counselor type, we will add additional respondents to reach saturation. Interviews will be conducted by a local interviewer in the language of the participant’s choosing (i.e., Kiswahili or English). Each interview will last approximately 1 h. Questions will focus on participants’ experiences using mobile technology to receive or conduct clinical supervision, including any perceived benefits and disadvantages of the approach. Interviews will also focus on perceived effectiveness of supervision via mobile technology.

#### Measures

Measures for Aim 3 constructs will be adapted from existing measures, prioritizing acceptability and feasibility measures already translated and used cross-culturally in the parent trial (i.e., BASIC) and other studies in globally. All adaptations to the usability measure will be made following established procedures to ensure common understanding of the construct [21].

##### Acceptability

The 4-item Acceptability of Intervention measure [41] will be adapted and used to assess lay counselor perspectives of mobile technology supervision acceptability. This brief, pragmatic measure has acceptable internal consistency (*α* = 0.85) and test-retest reliability (*r* = 0.80). Items will only be adapted slightly. “Intervention” will be replaced with the appropriate term for the type of supervision, as decided by community partners (e.g., “phone supervision was appealing” and “I welcome use of phone supervision)”.

##### Feasibility

The 4-item Feasibility of Intervention measure [41] will be adapted and used to assess lay counselor perspectives of mobile technology supervision feasibility. This brief, pragmatic measure has acceptable internal consistency (*α* = 0.89) and test-retest reliability (*r* = 0.88). Items will only be adapted slightly such that references to “intervention” are replaced with the appropriate term for the type of supervision (e.g., “phone supervision seems workable”).

##### Usability

The 10-item Intervention Usability Scale (IUS) [42] will be adapted and used to assess lay counselor perspectives of mobile technology supervision usability. The IUS has acceptable internal consistency (*α* = .83) [43]. Mentions of “intervention” will be replaced with the appropriate term for the type of supervision (e.g., “mobile phone supervision was easy to use)”.

#### Analyses

We will use descriptive statistics (mean, standard deviation, range) to understand counselor and supervisor ratings of acceptability, feasibility, and usability following mobile technology supervision. All quantitative analyses will be conducted using R [44]. Quantitative data will also be visualized to better illustrate cross-sector differences or outliers. All data will be stratified by sector (teachers in Education; CHVs in Health) to reflect perceptions of lay counselors situated within two different contexts. Given the sample size of 30 counselors for quantitative data, we will follow best practices for small samples and not conduct null hypothesis significance testing for differences in acceptability, feasibility, and usability. Qualitative data will be examined using the same thematic analysis procedure described in Aim 1. We will follow a QUAN → qual mixed methods approach for data explanation, using the embedded qualitative data to elaborate on or contextualize quantitative results [45].

#### Trial status

The Institutional Review Board (IRB) at the University of Washington has approved all study procedures. All procedures are under review at the Kenya Medical Research Institute’s IRB and will be subsequently submitted to Kenya’s National Commission for Science, Technology and Innovation, who will provide a permit for community entry. Recruitment and data collection for this study will begin in 2021.

## Discussion

This project will provide a launching point for future research on supervision and methods to engage stakeholders to design and tailor interventions and implementation supports to fit low-resourced contexts. This trial capitalizes on a naturally occurring phenomenon within an already funded trial (i.e., mobile technology use) and seeks to understand the potential of mobile technology supervision as a low-cost and accessible alternative. By conducting this research in a rural setting within a lower-middle income country, where barriers to use may be amplified as compared to other high-resourced settings in Kenya or the USA, we create opportunities to develop more creative and frugal implementation strategies and supports with high potential applicability for other lower-resourced settings [46]. Study findings may help inform the potential for greater reliance on lower-cost, existing mobile technology to provide clinical supervision across a variety of settings or inform use of HCD to tailor other implementation strategies. The ultimate goal is to generate guidance and evidence that can be applied beyond TF-CBT in Bungoma, Kenya, informing development of sustainable methods of clinical supervision across interventions and settings, including low-resourced settings in the USA.

There is increasing recognition of the potential of technology to address the mental health treatment gap. However, the majority of research has focused on client-facing applications of technology (i.e., applications directed toward mental health treatment and prevention) [47]. We are responsive to calls for investigating technology as a means of facilitating supervision and supporting mental health providers [6, 47–49] and do so within the context of existing technologies (i.e., text message and WhatsApp) for greater generalizability. We examine the potential to support mental health care providers by leveraging technologies that are already being used (as opposed to developing applications). This reduces cost and may increase generalizability to different contexts.

We engage lay providers and supervisors to inform how we leverage mobile technology supervision as an implementation strategy, thereby garnering local ownership and contextual understanding. This approach aims to increase likelihood of sustainment and acceptability. We are among the first to garner local expertise and engage stakeholders via HCD techniques in global mental health. To our knowledge, no other studies have applied HCD techniques to develop and refine implementation strategies in global mental health nor have any studies (global or USA) assessed participant perceptions of engaging in HCD techniques. This has implications for HCD use in global settings, as it was developed and is largely used in high income countries with Western cultural norms. Participant perceptions of HCD techniques in Kenya may shape their application in the future.

A logical follow-up study is to assess the effectiveness of mobile technology supervision in a randomized trial. Future work might also employ HCD to tailor supervision and other implementation strategies on a broader scale. This work could focus on other clinical interventions (e.g., substance abuse disorder treatment), contexts (e.g., rural areas in the USA; other LMIC), or implementation strategies [e.g., leadership and organizational interventions [50] or implementation facilitation [51]].

## Considerations and limitations

We selected our approach after consideration of alternative methods and designs. We have thought carefully about potential barriers and limitations to the selected approach. One barrier may be using HCD techniques in a culturally and contextually distinct setting. Given the power dynamics inherent in global research, all interviews and HCD workshops will be led by a Kenyan member of the research team. Any difficulties will be discussed and problem solved with local experts. This team has successfully, and collaboratively, made cultural and contextual modifications to methods in other work, such as in the parent trial. We also acknowledge the limitation in our pilot trial design that counselors are not randomized to mobile technology supervision, thereby limiting external validity and generalizability. Our sample may further limit generalizability, as counselors without WhatsApp compatible phones will be excluded from participation. We acknowledge that having a phone with WhatsApp compatibility may overlap with other important confounding variables, such as rurality or income. We feel this approach is appropriate to gather proof of concept for future research and given the limitations of this trial (i.e., nested within a larger randomized trial and a desire to work within existing resources).

## Supplementary Information

**Additional file 1.** CONSORT 2010 checklist of information to include when reporting a pilot or feasibility trial.

## Data Availability

Not applicable.

## Abbreviations

EBP: Evidence-based practice
HCD: Human-centered design
LMIC: Low- and middle-income countries
CONSORT: Consolidated Standards of Reporting Trials
BASIC: Building and Sustaining Interventions for Children
TF-CBT: Trauma-focused cognitive behavioral therapy
CHV: Community health volunteer
SOP: Standard operating procedure
IRB: Institutional Review Board
